# Contrast-free postoperative imaging of the pulmonary arteries: Intraindividual comparison of relaxation-enhanced angiography without contrast and triggering and time-resolved contrast-enhanced magnetic resonance angiography

**DOI:** 10.1016/j.jocmr.2026.102732

**Published:** 2026-04-17

**Authors:** Vanessa Schmidt, Lukas Goertz, Juliana Tristram, Robert Terzis, Kenan Kaya, Thorsten Gietzen, Kilian Weiss, David Maintz, Carsten Gietzen, Lenhard Pennig, Jan Paul Janssen

**Affiliations:** a Institute for Diagnostic and Interventional Radiology, Faculty of Medicine and University Hospital Cologne, University of Cologne, Cologne, Germany; b Department of Cardiology, Heart Center, Faculty of Medicine and University Hospital Cologne, University of Cologne, Cologne, Germany; c Philips Healthcare Germany, Hamburg, Germany

**Keywords:** REACT, 4D CE-MRA, Congenital heart disease, Pulmonary artery vasculature, Stent, Conduit

## Abstract

**Background:**

Flow-independent, three-dimensional isotropic non-contrast magnetic resonance angiography (MRA) such as relaxation enhanced angiography without contrast and triggering (REACT) are increasingly established as alternatives to contrast-enhanced MRA (CE-MRA) for assessment of the pulmonary arteries (PAs). However, their performance for dedicated postoperative assessment of the PAs in patients with congenital heart disease (CHD), particularly in the presence of different implant types, remains unclear.

**Methods:**

In this retrospective single-center study, 53 patients with CHD underwent clinically indicated cardiovascular magnetic resonance, including both four-dimensional (4D) CE-MRA and REACT at 1.5T. Three radiologists independently scored image quality (IQ) as well as motion and susceptibility artifacts on 5-point Likert scales and measured the diameters of the PAs [main (MPA), left (LPA), and right pulmonary artery (RPA)]. Subgroup analysis was performed for stents, conduit/patch/valve (CPV), and no implant.

**Results:**

Pooled across readers and PA segments, REACT achieved higher overall IQ than 4D CE-MRA (median 3.67 [3.00–4.17] vs 3.00 [2.33–3.33]; *p* < 0.001) and provided significantly better motion scores (*p* < 0.001), whereas susceptibility scores were comparable between techniques. The proportion of fully diagnostic studies (3/3 segments) was similar (REACT 77.4%, 41/53; 4D CE-MRA 83.0%, 44/53; McNemar, *p* = 0.38). Diameter measurements showed excellent inter-reader agreement (intraclass correlation coefficient ≈ 0.89–0.95) and minimal bias between techniques; only the RPA yielded slightly smaller diameters in REACT (mean difference −0.85 ± 1.51 mm, *p* < 0.001). In subgroup analysis, stented segments showed no IQ advantage of REACT (*p* > 0.99) with IQ being limited due to susceptibility artifacts in both 4D CE-MRA and REACT. In the CPV and the no-implant group, REACT yielded a one point higher median IQ score (both *p* = 0.002) and one point less impaired by motion artifacts (CPV: *p* < 0.001; no implant: *p* = 0.002), while both techniques provided very high shares of diagnostic IQ (defined as IQ ≥ 2; both > 90%; *p* > 0.99).

**Conclusion:**

REACT enables robust, contrast-free postoperative imaging of the PAs in patients with CHD with superior IQ and reduced motion artifacts compared to 4D CE-MRA, while maintaining highly reproducible diameter measurements. Stented segments remain a shared limitation.

## Graphical abstract

**Figure:**
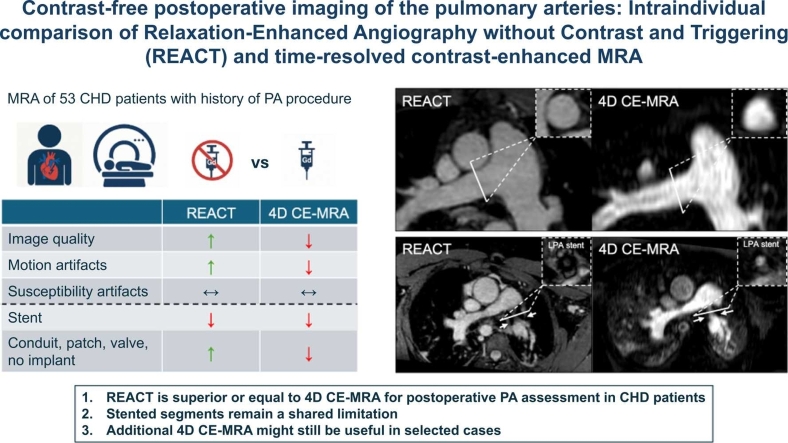

## 1 Introduction

Congenital pulmonary artery (PA) anomalies represent a heterogeneous spectrum of structural malformations of the cardiovascular system, ranging from incidental, asymptomatic findings to potentially fatal causes of sudden cardiac death. They may occur as isolated anomalies or in association with congenital heart disease (CHD). While certain abnormalities are diagnosed during childhood, others may remain undetected until they are incidentally identified in adulthood [1], [2].

Cardiovascular magnetic resonance (CMR) remains the key modality for imaging patients with congenital PA abnormalities and is essential in guiding the care of patients from initial diagnosis to long-term follow-up. Magnetic resonance angiography (MRA) is now widely established as the first-line non-invasive imaging approach for vascular assessment in various anatomical regions [3], [4]. Over the past decades, contrast-enhanced MRA (CE-MRA) has further evolved with the introduction of time-resolved techniques (four-dimensional [4D] CE-MRA), while consistently demonstrating high reliability in detecting vascular abnormalities such as vascular stenosis, dilatations, and other pathologies [3], [4], [5], [6], [7], [8]. However, performing 4D CE-MRA for the assessment of thoracic vascular pathologies is technically demanding and involves the use of contrast agents [3], [4], [6], [9]. Moreover, image quality (IQ) in CE-MRA is influenced by several factors, including precise synchronization of contrast injections and adequate patient compliance, as well as trade-offs between temporal and spatial resolution, and a high susceptibility to respiratory and cardiac motion may limit small-vessel delineation and the reproducibility of diameter measurements [6], [10].

To mitigate the limitations associated with gadolinium administration, such as patient preparation requiring intravenous access, the risk of anaphylactic reactions, restrictions during pregnancy, and others [11], [12], and to maintain high diagnostic IQ, non-CE-MRA sequences, such as two-dimensional (2D) and three-dimensional (3D) balanced steady-state free precession (bSSFP), have been developed to assess thoracoabdominal vessels [12], [13], [14], [15], [16]. In 2019, Yoneyama et al. [17] introduced relaxation-enhanced angiography without contrast and triggering (REACT), a non-contrast, flow-independent technique based on magnetization preparation and a 3D Dixon readout to enhance blood signal while suppressing background tissues allowing for a more robust non-CE-MRA over a larger field of view. Prior work has demonstrated excellent IQ and accurate vascular measurements of REACT across thoracic vascular territories, including the pulmonary vasculature, at 1.5T [12], [18], [19], [20], [21], [22], [23], [24]. Furthermore, Isaak et al. and Pennig et al. have suggested REACT as a contrast-free alternative for routine follow-up in patients with CHD, particularly for those undergoing repetitive imaging of the pulmonary vessels [21], [23].

Nevertheless, evidence focusing on the postoperative assessment of the PA remains limited. To our knowledge, no study has systematically differentiated performance by implant type, even though metallic stents, conduits, and patch materials can markedly degrade IQ due to susceptibility and off-resonance effects.

Therefore, we conducted an intraindividual comparison of IQ, artifacts, and diameter measurements between REACT and 4D CE-MRA in patients following PA procedures, with a particular emphasis on the impact of different implant types.

## 2 Methods

### 2.1 Study cohort

This single-center study was approved by the local institutional review board. Given its retrospective design, the board waived the requirement for written informed consent (reference number: 23–1,167-retro).

#### 2.1.1 Patient population

A retrospective review of the institutional imaging database at a tertiary care university hospital was conducted to identify patients with CHD and a history of PA procedures who underwent CMR between October 2018 and June 2024. Patients were eligible for inclusion if imaging had been performed according to a standardized protocol incorporating both REACT and 4D CE-MRA sequences. Exclusion criteria were the absence of either sequence. No exclusion criteria were specified for the type of CHD or the treatment procedure used.

### 2.2 Magnetic resonance imaging

All examinations were performed on a commercially available 1.5T MRI system (Philips Ingenia, Philips Healthcare, Best, The Netherlands) using a 28-channel coil for cardiac imaging. The CMR protocol comprised REACT (index test), 4D CE-MRA (clinical reference technique), 2D bSSFP breath-hold cine sequences in standard orientations (four-chamber, three-chamber, two-chamber, short-axis, axial, and aortic sinus), and a 2D phase contrast velocity measurement sequence for the aortic and pulmonary valves.

#### 2.2.1 Modified REACT

As described in previous publications, the “modified” REACT approach incorporates T2-preparation combined with a water-fat-selective Dixon (mDIXON XD, Philips Healthcare) readout [12], [19], [21], [22], [23]. Based on the assumption that background suppression with mDIXON XD in conjunction with T2 preparation is adequate in cardiovascular imaging, the use of an inversion-recovery prepulse was omitted [12], [21], [22]. Furthermore, to mitigate the effects of cardiac and respiratory motion, end-diastolic electrocardiogram-triggering and respiratory navigator-gating were added to the REACT sequence. These approaches represent a deviation from the original REACT sequence [17]. The 3D isotropic, flow-independent REACT sequence was obtained in coronal plane, encompassing the heart, focusing on the PA. Compressed SENSE (Philips Healthcare), combining principles of parallel imaging with compressed sensing [25], was applied with a factor of 9 for acceleration of image acquisition. The reconstruction was performed using standard hardware as provided by the CMR system’s manufacturer.

#### 2.2.2 Time-resolved contrast-enhanced MRA

Time-resolved (4D) CE-MRA was performed using a 3D T1-weighted spoiled gradient-echo sequence consisting of a single native pre-contrast frame followed by 12 contrast-enhanced dynamic frames with a temporal resolution of 1 s per frame. The gadolinium-based contrast agent Gadobutrol (Gadovist; Bayer HealthCare Pharmaceuticals, Leverkusen, Germany) was injected via an antecubital vein at a dose of 0.1 mL/kg body weight at a rate of 2 mL/s, followed by a 30 mL saline flush. Untriggered breath-holding was requested of patients during the image acquisition. Coronal data acquisition was initiated when bolus monitoring indicated a significant contrast arrival in the main PA. All 4D CE-MRA datasets were acquired with acceleration using sensitivity encoding (SENSE, Philips Healthcare), employing a factor of 3.

### 2.3 Image analysis

A commercially available image viewer (DeepUnity Diagnost, release 2.0.2.2, Dedalus Healthcare Systems Group, Bonn, Germany) was used for image analysis. All datasets were anonymized before analysis and presented in random order. Readers evaluated each dataset independently and were blinded to patient identifiers and clinical data as well as to the ratings of the other readers. Complete blinding to the imaging sequence was not feasible, because REACT and 4D CE‑MRA differ fundamentally in contrast mechanism and appearance. Two radiologists in training with 2 (R1) and 4 years (R2), and a board-certified radiologist with 6 years (R3) of experience in CMR performed subjective scoring and diameter measurements.

#### 2.3.1 Image quality

Subjective IQ was assessed for the following vascular regions during the same imaging sessions: the main pulmonary artery (MPA), as well as the left and right pulmonary artery (LPA; RPA). Assessment consisted of a 5-point ordinal scale based on three criteria:

1. Overall IQ: 1 = non-diagnostic (insufficient for clinical interpretation); 2 = poor (considerably reducing diagnostic confidence); 3 = moderate (acceptable for diagnostic purposes); 4 = good (allowing confident diagnosis); 5 = excellent (providing high diagnostic certainty).
2. Susceptibility artifacts: 1 = non-diagnostic (extremely high susceptibility preventing reliable evaluation); 2 = pronounced (susceptibility markedly impairing diagnostic interpretation); 3 = moderate (susceptibility moderately affecting evaluation); 4 = low (minimal effect on IQ); 5 = negligible (virtually no impact).
3. Motion artifacts: (including blurring, banding, pulsation, or reconstruction artifacts from parallel imaging): 1 = non-diagnostic (artifacts precluding interpretation); 2 = severe (strongly affecting IQ); 3 = moderate (artifacts with noticeable but acceptable effect); 4 = minor (minimal effect); 5 = none (artifacts absent).

For each patient, IQ was assessed on source images. Multiplanar reformations (MPR) were allowed to align the viewing plane and window/level adjustments were permitted. For 4D CE‑MRA, readers reviewed the time-resolved series (subtraction images) and based scoring and measurements on the frame with the most homogeneous pulmonary arterial enhancement. For REACT, scoring was based on the water-only reconstruction.

#### 2.3.2 Diameter measurement

For quantitative analysis, PA diameter measurements (inner-edge approach) were performed at the following three predefined landmarks: MPA (2 cm distal to the pulmonary valve), RPA (1 cm distal to the bifurcation), and LPA (1 cm distal to the bifurcation). The source images were analyzed using MPR derived from the 3D datasets.

Evaluation of the diagnostic PA segments of REACT and 4D CE-MRA was documented using the 5-point Likert scale ratings of the readers, whereby segments with an overall IQ score ≥2 were considered technically diagnostic. Vessels with non-diagnostic IQ were excluded from the quantitative analysis.

#### 2.3.3 Fat-water swapping artifacts and mistiming

As previously reported, fat-water swapping artifacts occur in REACT imaging, relying on DIXON-based water-fat separation to visualize the vascular system [22], [26], [27]. Due to the important role of accurate vessel visualization, the existence of fat-water swapping could result in significant diagnostic inaccuracies. Accordingly, the datasets were assessed for these artifacts, and they were defined as a loss of vascular signal on the water image accompanied by a corresponding hyperintense signal on the fat image.

Cases with suboptimal PA contrast in 4D CE-MRA, for example, due to a delayed scan start, were recorded.

### 2.4 Statistical analysis

Statistical analysis was conducted using GraphPad Prism (v. 10.4.2, GraphPad Software, San Diego, California) and R (v. 4.3.1, R Foundation for Statistical Computing, Vienna, Austria). Categorical variables were expressed as frequencies and corresponding percentages, and continuous data were reported as mean ± standard deviation (SD) or median (interquartile range [IQR]), after assessing normality (Shapiro-Wilk test and QQ-plots).

Paired comparisons between sequences were performed using the Student’s t-test or the Wilcoxon signed-rank test, as appropriate, based on patient-level aggregates (medians across readers for ordinal ratings; means across readers for diameter measurements). Effect sizes were reported as paired differences with 95% confidence intervals. Shares of diagnostic IQ (defined as IQ ≥ 2) were compared using McNemar’s test.

Inter-reader agreement for subjective IQ was evaluated using Kendall’s coefficient of concordance (W), with interpretation as follows: 0.01–0.2, slight; 0.21–0.4, fair; 0.41–0.6, moderate; 0.61–0.8, substantial; and 0.81–0.99, almost perfect. Inter-reader agreement of PA diameter measurements was assessed using intraclass correlation coefficient (ICC). Intersequence agreement of PA diameters was assessed using the ICC and further evaluated by Bland–Altman analysis.

Subgroup analyses by implant type used a prespecified hierarchical assignment to a single index segment. Patients were classified by the highest-priority implant (Stent > conduit/patch/valve [CPV] > None) and the most proximal vessel (MPA > LPA > RPA). Within each implant subgroup, paired comparisons between REACT and 4D CE-MRA were performed as described above to quantify the within-subgroup effect of sequence on IQ scores and diameter measurements. To assess whether the magnitude of these sequence effects differed by implant type, differences were calculated for each outcome and compared across the three implant subgroups using Kruskal–Wallis tests for ordinal outcomes and Welch’s analysis of variance for diameter differences, with appropriate post-hoc tests and multiplicity adjustment. Across all analyses, a two-tailed *p*-value of <0.05 was considered statistically significant.

To assess whether cases with suboptimal PA contrast in 4D CE-MRA affected the main findings, the primary comparisons were additionally repeated in an exploratory sensitivity analysis (post-hoc), excluding examinations with contrast mistiming. The same statistical tests, significance thresholds, and reporting formats as in the primary analysis were applied in the sensitivity analysis.

## 3 Results

### 3.1 Study population and baseline characteristics

**Fig. 1:**
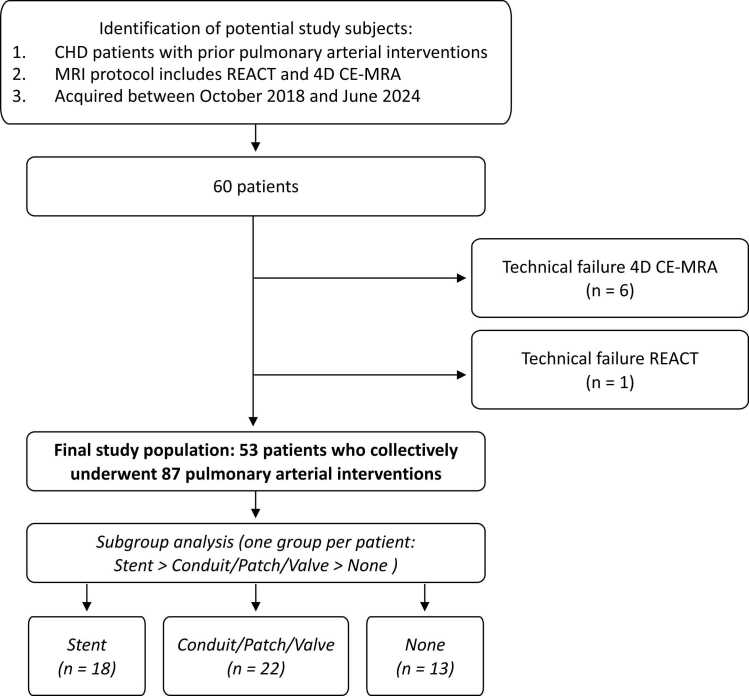
Workflow for inclusion and exclusion of cases. *4D CE-MRA* four-dimensional contrast-enhanced magnetic resonance angiography, *CHD* congenital heart disease, *MRI* magnetic imaging, *REACT* relaxation-enhanced angiography without contrast and triggering

Sixty patients were identified. Due to missing sequences or technical failures, seven patients were excluded. Therefore, 53 patients were included in the present study (Fig. 1).

The median age was 29 years [IQR 17–37]. Hypertension was the most frequent cardiovascular risk factor (17%; 9 of 53). Within the entire spectrum of CHD, tetralogy of Fallot was the most common disease (35.8%; 19 of 53), followed by pulmonary atresia (18.9%; 10 of 53), pulmonary valve stenosis (17%; 9 of 53), and transposition of the great arteries (11.3%; 6 of 53). Eighteen (34%; 18 of 53) patients were treated with at least one PA stent. In addition, 18 (34%; 18 of 53) patients underwent PA reconstruction or patch-angioplasty, and 15 (28.3%; 15 of 53) patients were treated with a right ventricular (RV)-PA conduit. Operative treatment was based on either an isolated procedure or a combination of multiple procedures. Detailed information about the baseline characteristics is summarized in Table 1.

**Table 1:** Study population and patients characteristics.

*Patient characteristics*	*Median [IQR]*	
Age (years)	29 [17–37]	
Height (cm)	167 [157–176]	
Weight (kg)	65 [49.8–78.5]	
BMI (kg/m^2^)	21.7 [19.8–27.3]	
Female sex, n (%)	27 (50.9)	
*Cardiovascular risk factors*	*n*	*%*
Hypertension	9	17.0
Diabetes mellitus	3	5.7
Dyslipidemia	4	7.5
Smoking	5	9.4
*Cardiac arrhythmia*	32	60.4
*Congenital heart disease*		
Tetralogy of Fallot	19	35.8
Pulmonary atresia	10	18.9
Pulmonary valve stenosis	9	17.0
Transposition of the great arteries	6	11.3
Truncus arteriosus	3	5.7
Double-outlet right ventricle	3	5.7
Aortic stenosis	2	3.8
Ebstein anomaly	1	1.9
*Associated septal defects*		
ASD only	5	9.4
VSD only	19	35.8
ASD and VSD	3	5.7
*Operations (pulmonary arteries)*		
Stent	18	34.0
Reconstruction/patch	18	34.0
RV-PA conduit	15	28.3
Pulmonary valve replacement	10	18.9
Valvotomy/commissurotomy/valvuloplasty	12	22.6
Pulmonary artery balloon angioplasty	11	20.8
Anastomosis	3	5.7
*Subgroup analysis by implant type* (one index segment per patient stent > conduit/patch/valve > none)		
Stent	18	34.0
Conduit/patch/valve	22	41.5
None	13	24.5

*ASD* atrial septum defect, *BMI* body mass index, *IQR* interquartile range, *PA* pulmonary artery, *RV* right ventricular, *VSD* ventricular septum defect

Categorical variables were expressed as frequencies and corresponding percentages, and continuous data were reported as mean ± standard deviation (SD) or median (interquartile range [IQR]).

### 3.2 Image quality evaluation

#### 3.2.1 Image acquisition

Including image reconstruction, the total duration of the REACT examination was 5:47 min [4:42–8:56], compared to 4:07 min [3:15–6:54] (*p* = 0.01) for the complete 4D CE-MRA protocol (including native acquisition and bolus tracking).

An overview of the acquisition parameters for both sequences is provided in Table 2.

**Table 2:** Scan parameters.

Scan parameters	4D CE-MRA	REACT
K-space trajectory	Cartesian	Cartesian
Acquisition orientation	Coronal	Coronal
Acquired voxel size (mm)	1.49 × 1.49 × 4	1.70 × 1.70 × 1.70
Acquisition matrix size	268 × 268 × 50	236 × 299 × 200
Reconstructed voxel size (mm)	0.93 × 0.93 × 2.00	0.79 × 0.79 × 0.85
Field of view (FH × RL × AP) (mm)	400 × 400 × 100	400 × 508 × 170
T2 preparation	N/a	50 ms, refocusing pulses: 4
Repetition time (ms)	2.8	6.3
Echo time (1/2) (ms)	1.05	1.81/4.0
Flip angle	30°	15°
k-space lines per heartbeat	N/a	35
Acceleration factor	SENSE 3	Compressed SENSE 9
Temporal resolution (s)	1	N/a
Dynamic scans	12	N/a
Gating window	N/a	5 mm
Subtraction	CE—native	N/a
Image reconstruction	Real time	Immediate
Total acquisition time (min)	04:07 [03:15–06:54]	05:47 [04:42–08:56]

*4D CE-MRA* four-dimensional contrast-enhanced magnetic resonance angiography, *AP* anterior posterior, *CE* contrast-enhanced, *FH* feet head, *N/a* not available, *REACT* relaxation-enhanced angiography without contrast agent and triggering, *RL* right-left, *SENSE* sensitivity encoding

Categorical variables were expressed as frequencies and corresponding percentages, and continuous data were reported as mean ± standard deviation (SD) or median (interquartile range [IQR]).

#### 3.2.2 Subjective image quality

**Fig. 2:**
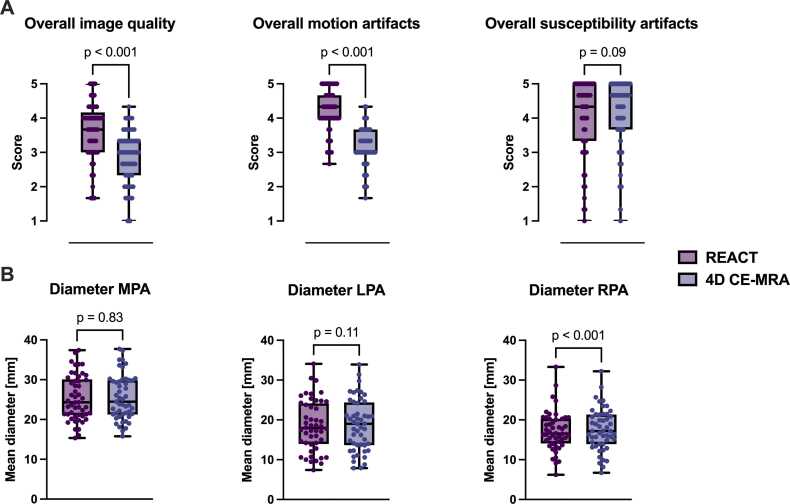
Overall subjective ratings, median of three readers and mean of three segments (A) and segment-wise mean diameter as mean of three readers (B). *4D CE-MRA* four-dimensional contrast-enhanced magnetic resonance angiography, *LPA* left pulmonary artery, *MPA* main pulmonary artery, *REACT* relaxation-enhanced angiography without contrast and triggering, *RPA* right pulmonary artery

**Fig. 3:**
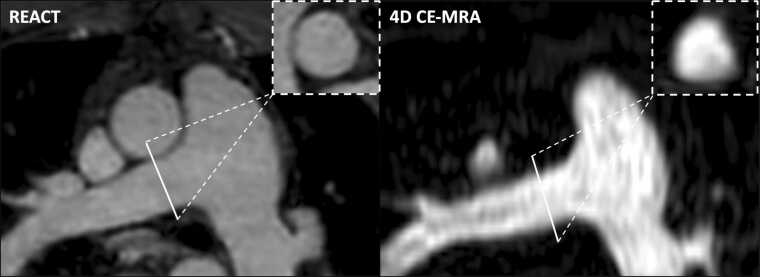
Comparison of REACT and 4D CE-MRA in a 43-year-old male patient with valvuloplasty of the pulmonary valve. Source images (axial plane angulated to the pulmonary artery bifurcation) from REACT (water-only) and 4D CE-MRA are presented. REACT provides superior image quality in all segments. Axial reformations of the right pulmonary artery are shown in the insets to demonstrate the reduced blurring observed in REACT. *4D CE-MRA* four-dimensional contrast-enhanced magnetic resonance angiography, *REACT* relaxation-enhanced angiography without contrast and triggering

A total of 106 datasets (53 REACT; 53 4D CE-MRA) were assessed independently by the three readers. Combined for all three readers and all three PA segments (MPA, LPA, and RPA), REACT achieved significantly higher IQ compared to 4D CE-MRA (3.67 [3.00–4.17] vs 3.00 [2.33–3.33]; *p* < 0.001). Additionally, REACT showed significantly better motion scores in all vessels (*p* < 0.001). No significant difference was observed between both techniques regarding susceptibility artifacts. Fig. 2 presents the overall subjective ratings, pooled across the three readers and the three segments (A), as well as average vessel diameters (B) derived from the measurements of the three readers. Fig. 3 shows an example image that illustrates the differences in IQ and motion artifacts.

The overall concordance between readers was excellent, as reflected by Kendall’s coefficients ranging from W = 0.64 (overall motion artifacts) to W = 0.82 (overall susceptibility artifacts). The corresponding coefficients of all individual ratings are summarized in Table 3.

**Table 3:** Subjective ratings and shares of diagnostic image quality pooled for all readers.

	REACT	4D CE-MRA	Median of paired differences (REACT − 4D CE-MRA) (95%-CI)	*p* (Wilcoxon)	Kendall’s W	
	Median [IQR]	Median [IQR]			REACT	4D CE-MRA
*Image quality*						
Overall	3.67 [3.00–4.17]	3.00 [2.33–3.33]	0.67 (–0.67 to 1.00)	**<0.001**	0.78	0.73
MPA	4.00 [3.00–4.00]	3.00 [2.00–3.00]	1.00 (1.00–1.00)	**<0.001**	0.77	0.68
LPA	4.00 [3.00–4.00]	3.00 [2.00–4.00]	0.00 (0.00–1.00)	**<0.001**	0.81	0.82
RPA	4.00 [3.00–4.00]	3.00 [2.00–4.00]	1.00 (1.00–1.00)	**<0.001**	0.75	0.68
*Motion artifacts*						
Overall	4.33 [4.00–4.67]	3.00 [3.00–3.67]	1.00 (1.00–1.33)	**<0.001**	0.64	0.62
MPA	4.00 [4.00–5.00]	3.00 [3.00–4.00]	1.00 (1.00–1.00)	**<0.001**	0.63	0.62
LPA	4.00 [4.00–5.00]	3.00 [3.00–4.00]	1.00 (1.00–1.00)	**<0.001**	0.68	0.69
RPA	4.00 [4.00–5.00]	3.00 [3.00–3.50]	1.00 (1.00–1.00)	**<0.001**	0.62	0.54
*Susceptibility artifacts*						
Overall	4.33 [3.33–5.00]	4.67 [3.67–5.00]	0.00 (0.00–0.00)	0.092	0.82	0.74
MPA	4.00 [3.00–5.00]	4.00 [3.00–5.00]	0.00 (0.00–0.00)	0.952	0.79	0.76
LPA	5.00 [3.50–5.00]	5.00 [4.00–5.00]	0.00 (0.00–0.00)	0.266	0.87	0.72
RPA	5.00 [3.00–5.00]	5.00 [4.00–5.00]	0.00 (0.00–0.00)	0.274	0.82	0.76
*Shares of image quality ≥2*	REACT diagnostic [n, %]	4D CE-MRA diagnostic [n, %]	Discordant b/c	*p* (McNemar)		
Overall (3/3)	41/53, 77.4%	44/53, 83.0%	1/4	0.375		
MPA	48/53, 90.6%	48/53, 90.6%	2/2	1.000		
LPA	48/53, 90.6%	49/53, 92.5%	0/1	1.000		
RPA	48/53, 90.6%	50/53, 94.3%	1/3	0.625		

*4D CE-MRA* four-dimensional contrast-enhanced magnetic resonance angiography, *IQR* interquartile range, *LPA* left pulmonary artery, *MPA* main pulmonary artery, *REACT* relaxation-enhanced angiography without contrast and triggering, *RPA* right pulmonary artery

p-values < 0.05 were considered statistically significant and are indicated in bold.

Categorical variables were expressed as frequencies and corresponding percentages, and continuous data were reported as mean ± standard deviation (SD) or median (interquartile range [IQR]).

#### 3.2.3 Pulmonary artery diameter measurements

**Fig. 4:**
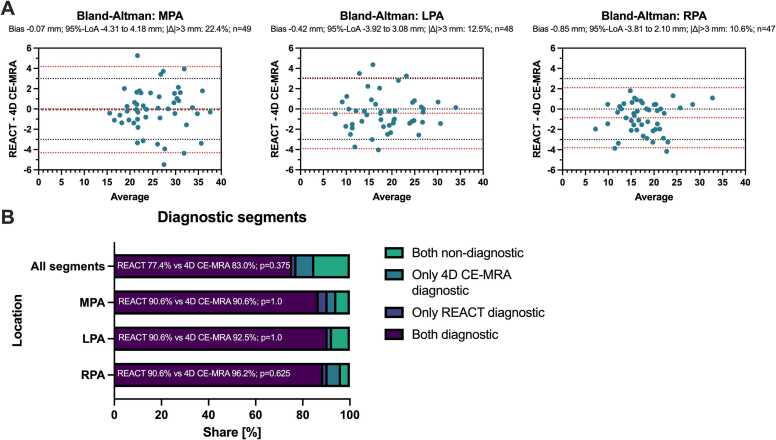
Bland–Altman plots (A) illustrate differences in diameter measurements between REACT and 4D CE-MRA. Stacked bar plots (B) show the shares of non-diagnostic studies and segments. *4D CE-MRA* four-dimensional contrast-enhanced magnetic resonance angiography, *LPA* left pulmonary artery, *MPA* main pulmonary artery, *REACT* relaxation-enhanced angiography without contrast and triggering, *RPA* right pulmonary artery

Six PA segments were not assessable on either REACT or 4D CE-MRA due to susceptibility artifacts or congenital or postoperative absence of the respective vascular structures. Due to segment-wise non-diagnostic IQ, diameter analysis included 49 cases for MPA, 48 for LPA, and 47 for RPA. In general, vessel diameters of the PA were largely similar, with a tendency toward smaller diameters in REACT. Only for the RPA, a significant difference was observed (*p* < 0.001). Overall, the inter-reader agreement between REACT and 4D CE-MRA was excellent for all diameter measurements, with ICC values ranging from approximately 0.89 to 0.95. Diameter measurements and Bland–Altman plots are presented in Table 4 and Fig. 4A, respectively.

**Table 4:** Diameter measurements and inter-reader agreement between all readers.

Segment	REACT diameter mean±SD (mm)	4D CE-MRA diameter mean±SD (mm)	*p* (t-test)	Bias (REACT − 4D CE-MRA) (mm)	LoA (mm)	Outliers >3 mm [n, %]	Inter-reader ICC (95% CI)	
							REACT	4D CE-MRA
MPA (n = 49)	25.59±5.66	25.56±5.63	0.83	−0.07	−4.31 to 4.18	11, 22.4%	0.89 (0.84–0.93)	0.91 (0.87–0.94)
LPA (n = 48)	18.49±6.41	18.91±6.36	0.11	−0.42	−3.92 to 3.08	6, 12.5%	0.95 (0.92–0.97	0.93 (0.90–0.96)
RPA (n = 47)	17.29±5.00	18.15±4.86	**<0.001**	−0.85	−3.81 to 2.10	5, 10.6%	0.95 (0.90–0.97)	0.91 (0.85–0.94)

*4D CE-MRA* four-dimensional contrast-enhanced magnetic resonance angiography, *REACT* relaxation-enhanced angiography without contrast agent and triggering, *MPA* main pulmonary artery, *LPA* left pulmonary artery, *RPA* right pulmonary artery, *LoA* limits of agreement, *SD* standard deviation, *ICC* intraclass correlation coefficient

p-values < 0.05 were considered statistically significant and are indicated in bold.

Categorical variables were expressed as frequencies and corresponding percentages, and continuous data were reported as mean ± standard deviation (SD) or median (interquartile range [IQR]).

Considering diagnostic segments (IQ ≥ 2), 4D CE-MRA showed slightly higher rates compared to REACT, but absolute differences were small and not significant (Table 3; Fig. 4B). For example, the share of examinations in which all three PA segments were rated as diagnostic was similar for both techniques (REACT 77.4% [41/53] vs 4D CE-MRA 83.0% [44/53]; *p* = 0.38).

#### 3.2.4 Fat-water swapping artifacts and mistiming

Overall, fat-water swapping artifacts occurred in 24 of the 53 cases (45.3%). Assessment revealed these artifacts in the left subclavian artery (37.5%; 9 of 53), aortic arch (16.7%; 4 of 53), PA system (37.5%; 9 of 53), pulmonary veins (8.3%; 2 of 53), and inferior vena cava (16.7%; 4 of 53).

The presence of fat-water swapping did not significantly affect the evaluation of vascular implants. The artifacts were observed in 8 of 18 cases with stents (44.4%), 10 of 22 cases with CPV (45.5%), and 6 of 13 cases without any implants (46.2%).

In 12 of 53 patients (22.6%), 4D CE-MRA demonstrated reduced PA enhancement due to mistiming of the acquisition relative to contrast passage. Sensitivity analysis excluding these cases yielded consistent results (Supplementary Tables S1 and S2).

### 3.3 Assessment of vascular implants

Regarding IQ, stented index segments showed no significant difference between the REACT and 4D CE-MRA sequence (median difference: 0 [−0.25 to 0]; *p* > 0.99) and remained limited by susceptibility artifacts (0 [−1 to 0]; *p =* 0.76). However, motion artifacts showed higher median differences in REACT compared to 4D CE-MRA (+1 [0–2]; *p* = 0.005). The share of diagnostic IQ in stented segments was slightly higher for 4D CE-MRA (66.7%; 12 of 18) than for REACT (50.0%; 9 of 18), but this difference was not significant (*p =* 0.25). Of note, 3 of 18 of the stented index segments had diagnostic IQ values in 4D CE-MRA but not in REACT, and 6 of 18 of the stented index segments did not provide diagnostic IQ values in either sequence.

In both CPV and no implants subgroup, REACT provided significant higher IQ (CPV: +1 [0–2]; *p* = 0.002 / None: +1 [0.5–2]; *p* = 0.002) and fewer motion artifacts (CPV: +1 [1, 2]; *p <* 0.001 / None: +1 [0.5–2]; *p* = 0.002). Regarding the diagnostic segments in CPV subgroup, both techniques showed high rates with 100% for REACT (22 of 22) and 95.5% for 4D CE-MRA (21 of 22).

In the across-group analyses, only median IQ differences (REACT − 4D CE-MRA) differed significantly between implant subgroups (Kruskal–Wallis *p* < 0.001). Post-hoc Dunn’s tests showed significantly smaller median IQ differences in the stent subgroup compared to both the CPV subgroup (*p* = 0.02) and the no-implant subgroup (*p* < 0.001), whereas CPV and no-implant subgroups did not differ (*p* = 0.59). For the corresponding differences in motion scores, susceptibility scores, and diameter measurements, no significant across-group differences were observed.

**Fig. 5:**
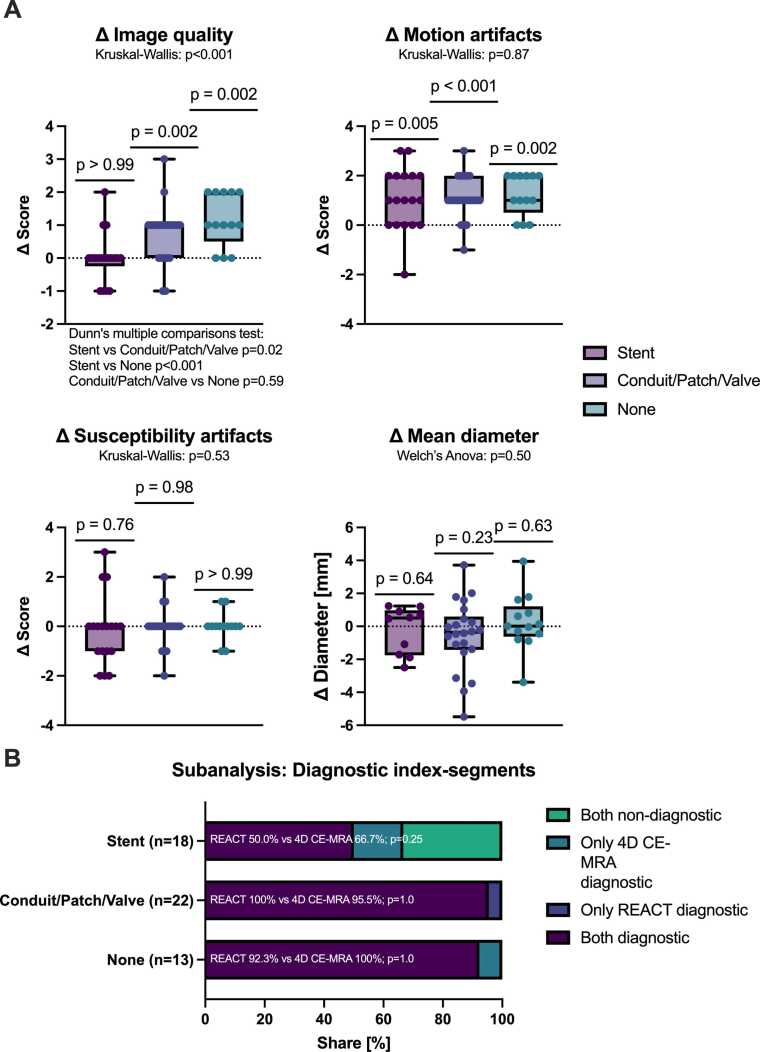
Box plots with whiskers (A) display the differences between REACT and 4D CE-MRA for the three subgroups regarding scores in image quality, motion artifacts, and susceptibility artifacts as well as diameter measurements. The *p*-values above each box plot indicate differences within each group, while the *p*-values under each heading indicate across-group differences. Stacked bar plots (B) show the shares of index segments with diagnostic and non-diagnostic image quality. *4D CE-MRA* four-dimensional contrast-enhanced magnetic resonance angiography, *REACT* relaxation-enhanced angiography without contrast agent and triggering

**Fig. 6:**
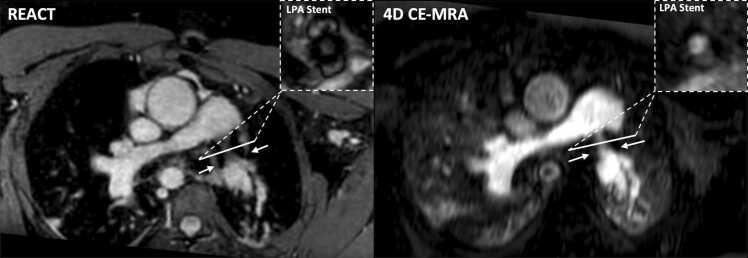
Comparison of REACT and 4D CE-MRA in a 29-year-old male patient with a stent in the left pulmonary artery (LPA) following multiple surgical corrections for tetralogy of Fallot. Source images (axial plane angulated to the pulmonary artery bifurcation) from REACT (water-only) and 4D CE-MRA are presented. Susceptibility artifacts caused by the metallic stent (white arrows), along with reduced intraluminal signal, are evident in both sequences. Axial reformations of the LPA stent are shown in the insets. 4D CE-MRA demonstrates superior image quality in the stented LPA compared to REACT. However, REACT exhibits superior image quality and fewer motion artifacts in the other segments. *4D CE-MRA* four-dimensional contrast-enhanced magnetic resonance angiography, *REACT* relaxation-enhanced angiography without contrast and triggering

**Fig. 7:**
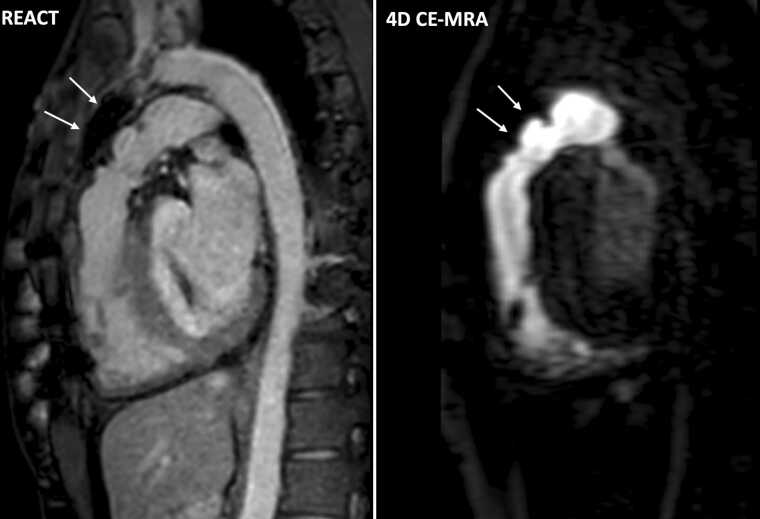
Comparison of REACT and 4D CE-MRA in a 23-year-old female patient with a right ventricular to pulmonary artery (RV-PA) conduit following Ross operation for critical valvular aortic stenosis. Source images (sagittal plane angulated to the RV-PA conduit) from REACT (water-only) and 4D CE-MRA are presented. Susceptibility artifacts mainly caused by the adjacent metallic reconstruction of the aortic valve (white arrows). REACT demonstrates superior image quality compared to 4D CE-MRA. *4D CE-MRA* four-dimensional contrast-enhanced magnetic resonance angiography, *REACT* relaxation-enhanced angiography without contrast and triggering

Subgroup characteristics and detailed IQ and artifact score comparisons (within-group and across-group) by implant type are summarized in Table 5 and Fig. 5. Image examples are shown in Figs. 6 and 7. Sensitivity analysis excluding CE-MRA mistiming cases yielded consistent results (Supplementary Table S3).

**Table 5:** Subgroup analysis by implant type.

Group	n	Δ IQ median [IQR]	*p* (vs 0)	Δ Motion median [IQR]	*p* (vs 0)	Δ Susceptibility median [IQR]	*p* (vs 0)
Stent	18	0[−0.25 to 0]	>0.99	1 [0–2]	**0.005**	0[−1 to 0]	0.76
CPV	22	1[0–1]	**0.002**	1 [1, 2]	**<0.001**	0 [0–0]	0.98
None	13	1[0.5–2]	**0.002**	1 [0.5–2]	**0.002**	0 [0–0]	>0.99
*Across-groups p (KW)*		<0.001*		*0.87*		*0.53*	
							
	n	REACT diameter mean±SD (mm)	4D CE-MRA diameter mean±SD (mm)	*p* (paired t)	Bias (REACT − 4D CE-MRA) (mm)	LoA (mm)	Outliers >3 mm [n, %]
Stent	10	14.72±6.07	14.94±5.51	0.64	−0.22	−3.00 to 2.56	0, 0%
CPV	22	23.72±5.78	24.28±5.83	0.23	−0.56	−4.68 to 3.57	5, 22.7%
None	13	29.67±4.65	29.43±4.92	0.63	0.24	−3.11 to 3.58	2, 15.4%
*Across-groups p (Welch’s ANOVA)*					*0.50*		
							
	n	REACT diagnostic [n, %]	4D CE-MRA diagnostic [n, %]	Discordant b/c	*p* (McNemar)		
Stent	18	9, 50.0%	12, 66.7%	0/3	0.25		
CPV	22	22, 100%	21, 95.5%	1/0	1.0		
None	13	12, 92.3%	13, 100%	0/1	1.0		

*4D CE-MRA* four-dimensional contrast-enhanced magnetic resonance angiography, *CPV* conduit/patch/valve, *IQ* image quality, *IQR* interquartile range, *REACT* relaxation-enhanced angiography without contrast agent and triggering, *LoA* limits of agreement, *ANOVA* analysis of variance, *KW* Kruskal-Wallis

Within group p-values < 0.05 were considered statistically significant and are indicated in bold. Across group p-values are indicated in italics and p-values < 0.05 were considered statistically significant and are indicated in bold-italics.

Categorical variables were expressed as frequencies and corresponding percentages, and continuous data were reported as mean ± standard deviation (SD) or median (interquartile range [IQR]).

* Dunn's multiple comparisons test: stent vs conduit/patch/valve *p* = 0.02; stent vs none *p <* 0.001; conduit/patch/valve vs none *p* = 0.59

## 4 Discussion

This retrospective single-center study compared REACT and 4D CE-MRA for postoperative imaging of the PAs in patients with CHD, with particular focus on different implant types. The main findings of this study are the following: (1) REACT provided higher IQ scores and markedly reduced motion artifacts in all PA segments than 4D CE-MRA, while susceptibility artifacts did not differ significantly between techniques. (2) Diameter measurements showed excellent inter-reader agreement and very good intersequence agreement, with only a small bias toward smaller RPA diameters on REACT. (3) Segment-wise shares of diagnostic IQ were comparable between REACT and 4D CE-MRA without significant differences. (4) In subgroup analyses, stented segments showed no IQ advantage of REACT and remained limited by susceptibility, whereas CPV and no-implant segments benefited from higher IQ and fewer motion artifacts on REACT with very high shares of diagnostic IQ in both sequences.

These results are in line with the findings of Pennig et al., who demonstrated higher IQ values for modified REACT in PAs and veins compared to 4D CE-MRA, along with high diagnostic quality for thoracic vascular assessment in adult patients with CHD [21]. Furthermore, Isaak et al. [23], [24], who investigated REACT in children and adults with CHD, emphasized the limited comparability between young children with CHD and adult cohorts due to complex cardiovascular hemodynamics and flow-related effects. Overall, REACT demonstrated high IQ and diagnostic quality comparable to CE-MRA, even in this young pediatric cohort [23].

In line with previous works comparing non-CE-MRA techniques, such as 3D bSSFP [10], [15] or modified REACT [21], to (4D) CE-MRA, REACT yielded precise and reliable vessel diameter measurements similar to those obtained with CE-MRA [23]. Pennig et al. previously reported significantly larger measured pulmonary vessel diameters in 4D CE-MRA, likely due to motion artifacts, physiological variability in PA size during different heart cycles, and strong pulsation artifacts (e.g., MPA: mean difference 0.41 mm). These effects were especially apparent in patients with CHD, as tetralogy of Fallot, where pulsatile flow within the PA may lead to overestimation of diameter in 4D CE-MRA [21], [28], [29], [30], [31], [32]. This might as well explain the tendency toward larger diameters in 4D CE-MRA compared to REACT in the present study, with significant differences exclusively for the RPA (mean difference 0.85 mm, *p* < 0.001). Given the Bland–Altman limits and the overall excellent agreement between readers and sequences, it is unlikely that this bias is clinically relevant; however, the same techniques should be used whenever possible in follow-up examinations.

Furthermore, the present data indicate that REACT provides comparable diagnostic IQ to 4D CE-MRA in detecting PA pathologies. Among the discordant cases, a small number of cases were noted in which 4D CE-MRA provided diagnostic IQ while REACT was non-diagnostic. This finding suggests that, in individual cases, the additional use of 4D CE-MRA may have diagnostic benefits. However, both techniques have inherent limitations, and in selected equivocal cases where imaging findings may affect therapeutic decision-making, additional computed tomography angiography (CTA) or digital subtraction angiography (DSA) may be warranted.

Besides susceptibility and motion artifacts, fat-water swapping artifacts are frequently reported as a common occurrence in Dixon-based imaging, such as REACT [12], [19], [22], [33]. In our study, fat-water swapping artifacts were observed in 45.3% of the cases, slightly higher than previously reported by Gietzen et al. with 40% of examinations [22] and Isaak et al. with 16%–33% of patients after surgery [23], [24]. Consistent with these studies mentioned above and prior investigations of REACT for cervical artery imaging, these artifacts were predominantly observed in the left subclavian artery [34], [35], [36]. In our study, fat-water artifacts occurred most frequently with a percentage of 37.5% (9 of 53) each in the left subclavian artery and the PA system. Importantly, we did not observe an impact on the assessment of implants or on the ability to detect relevant PA pathology, but careful review of both water and fat images remains mandatory.

However, performing 4D CE-MRA remains technically challenging, as optimal image acquisition requires precise coordination of contrast administration, accurate timing of data collection, and patient compliance with breath-holding instructions. 4D CE-MRA has been applied, reducing reliance on accurate bolus timing during contrast passage [6], [21]. Nevertheless, this study observed suboptimal timing of contrast bolus in 12 out of 53 cases (22.6%), resulting in a subsequent loss of IQ. Importantly, the sensitivity analysis excluding these cases showed unchanged direction and magnitude of the main findings, suggesting that the observed image-quality advantage of REACT is not solely driven by suboptimal contrast timing in 4D CE‑MRA.

The currently available data on postoperative evaluation of the PA, especially in cases involving vascular implants and altered anatomical configurations, remain limited. Assessment of complex vascular reconstructions in patients with CHD, including stents, conduits, or patch repairs, remains challenging due to altered anatomy and susceptibility to motion and metal-related artifacts. The increasing use of intravascular stents for the treatment of stenosis has raised the question of whether stent patency can be reliably assessed using MRA. The accuracy of CE-MRA in this context has been limited in previous studies due to susceptibility artifacts, which vary by stent material, geometry, and magnetic field strength [37], [38], [39].

Consistent with the above-mentioned studies, the results of this study showed a limited IQ of the stented PA segments equally in both REACT and 4D CE-MRA, while REACT clearly outperformed 4D CE-MRA in terms of IQ and motion in the non-stented segments. In 3 of 18 individual cases, 4D CE-MRA showed diagnostic IQ in stented PA segments, while REACT was non-diagnostic. In these instances, 4D CE-MRA should not be omitted, as it might provide additional diagnostic value for evaluating the stented segment. However, it should be noted that in these cases, 4D CE-MRA also showed limited IQ, and in 6 of 18 cases, neither sequence was able to achieve a diagnostic IQ of the stented segments. Ultimately, neither sequence is suitable for dedicated stent assessment. However, several studies demonstrated the superiority of CTA or DSA over CE-MRA for the assessment of stented vessels; therefore, complementary imaging may be considered and discussed in consensus [40], [41], [42], [43].

Previous investigations have emphasized the value of MRA, with or without contrast enhancement, for detailed assessment of PA. These techniques are especially recommended as a diagnostic modality before intervention in patients with dysfunctional right ventricular outflow tract, RV-PA conduits, or surgical bioprosthetic [44], [45], [46]. Our findings show that REACT achieved significantly higher IQ and motion scores in patient subgroups with CPV, while maintaining very high shares of diagnostic IQ (100% for REACT [22 of 22] vs 95.5% for 4D CE-MRA [21 of 22]). This observation supports the clinical applicability of REACT in ensuring reliable pre- and postoperative cardiovascular imaging.

The modified REACT represents a reliable imaging technique for evaluating postoperative anatomy in patients with CHD and can serve as a contrast-free first-line option, providing high-quality visualization of the PA tree and reliable diameter measurements, while avoiding repeated gadolinium exposure and mitigating issues of bolus mistiming. Conversely, it is recommended that radiologists carefully review the acquired images of patients with stented PA segments and, if necessary, supplement them with 4D CE-MRA. This approach may offer additional diagnostic value in selected cases.

## 5 Limitations

There are some limitations in this study. First, the findings were based on a single center and were retrospectively analyzed. Second and most paramount, the sample size was limited to 53 patients and the heterogeneity of the study population, which comprises patients with various types of CHD and postoperative alterations. However, the sample size was larger than in similar studies [21]. Third, 4D CE-MRA cannot be regarded as the true gold standard in the postoperative CHD setting, particularly in the presence of metallic implants or suboptimal contrast timing. Although DSA and CTA would have provided more appropriate reference standards for vascular assessment and in-stent evaluation, their use was not feasible in this retrospective study because both involve ionizing radiation and were not clinically indicated in this patient cohort. Therefore, this study does not allow assessment of the diagnostic accuracy of REACT but rather represents a comparative evaluation of two competing imaging techniques in the postoperative CHD setting. Furthermore, no comparison to other respiratory-gated non-CE-MRA sequences, such as 3D bSSFP, was conducted in this study. Fourth, suboptimal timing of contrast arrival limited several 4D CE-MRA examinations. While this reflects an important real-world limitation of the technique, it may have biased IQ comparisons in favor of REACT. However, post-hoc sensitivity analysis excluding these cases showed that the main findings remained unchanged. To reduce the risk of misjudgment, readers with several years of MRI experience were consulted for the evaluation. Fifth, intra-reader (test-retest) agreement was not assessed because each reader rated each dataset once. Sixth, complete blinding to the imaging sequence was not feasible, because REACT and 4D CE‑MRA differ fundamentally in contrast mechanism and appearance. This may have affected reader's judgment and thereby biased the subjective IQ assessments. Finally, subgroup analyses by implant type were based on relatively small numbers and should be regarded as exploratory. The study was not powered for detailed implant-specific outcome analyses.

## 6 Conclusion

REACT allows for robust, contrast-free postoperative imaging of the PAs in patients with CHD with superior IQ and reduced motion artifacts compared to 4D CE-MRA, while maintaining highly reproducible diameter measurements. Findings remained consistent after excluding 4D CE‑MRA examinations affected by bolus mistiming. However, stented segments remain a limitation of both techniques, as neither reliably evaluates the stent lumen. Although 4D CE-MRA may offer complementary diagnostic value in selected cases, additional CTA or DSA should be considered in equivocal cases or when imaging findings may affect therapeutic decision-making.

## Funding

Not funded.

## Author contributions

**Lenhard Pennig:** Writing – review & editing, Validation, Supervision, Project administration, Conceptualization. **Vanessa Schmidt:** Writing – original draft, Visualization, Methodology, Investigation, Formal analysis, Data curation, Conceptualization. **Jan Paul Janssen:** Writing – original draft, Visualization, Software, Project administration, Methodology, Investigation, Formal analysis, Data curation, Conceptualization. **Kilian Weiss:** Writing – review & editing, Validation, Investigation. **Thorsten Gietzen:** Writing – review & editing, Validation, Investigation. **Carsten Gietzen:** Writing – review & editing, Validation, Supervision, Project administration, Investigation, Data curation, Conceptualization. **David Maintz:** Writing – review & editing, Supervision, Resources. **Juliana Tristram:** Writing – review & editing, Validation, Investigation. **Lukas Goertz:** Writing – review & editing, Methodology, Investigation, Formal analysis, Data curation. **Kenan Kaya:** Writing – review & editing, Validation, Investigation, Conceptualization. **Robert Terzis:** Writing – review & editing, Validation, Investigation.

## Ethics approval and consent

This single-center study was approved by the local institutional review board. Given its retrospective design, the board waived the requirement for written informed consent (reference number: 23–1,167-retro).

## Declaration of Generative AI and AI-assisted technologies in the writing process

During the preparation of this work, the author(s) used ChatGPT (Version 5.1; OpenAI) and DeepL Translate (DeepL SE) to enhance the structure and quality of the language. After using this tool/service, the author(s) reviewed and edited the content as needed and take(s) full responsibility for the content of the publication.

## Declaration of competing interests

The authors declare the following financial interests/personal relationships which may be considered as potential competing interests: David Maintz: Speaker’s bureau, Philips Healthcare. Lenhard Pennig: Speaker’s bureau, Philips Healthcare. Speaker’s bureau, Guerbet GmbH. Kilian Weiss: Employee, Philips GmbH. The remaining authors disclose no relevant relationships.

## Data Availability

The datasets used and/or analyzed during the current study are available from the corresponding author on reasonable request.
