# Supplementary material for: Contrast-free postoperative imaging of the pulmonary arteries: Intraindividual comparison of relaxation-enhanced angiography without contrast and triggering and time-resolved contrast-enhanced magnetic resonance angiography
Source: J Cardiovasc Magn Reson. 2026 Apr 17;28(1):102732. doi: 10.1016/j.jocmr.2026.102732 (PMC13246309; doi:10.1016/j.jocmr.2026.102732)
Supplement: Supplementary file 1 — Supplementary material [file mmc1.docx]

***Supplementary Data***

**Contrast-free postoperative imaging of the pulmonary arteries: Intraindividual comparison of Relaxation-Enhanced Angiography without Contrast and Triggering (REACT) and time-resolved contrast-enhanced MRA**

Vanessa Schmidt^1^, Lukas Goertz^1^, Juliana Tristram^1^, Robert Terzis^1^, Kenan Kaya^1^, Thorsten Gietzen^2^, Kilian Weiss^3^, David Maintz^1^, Carsten Gietzen^1^, Lenhard Pennig^1^, Jan Paul Janssen^1^*

^1^Institute for Diagnostic and Interventional Radiology, Faculty of Medicine and University Hospital Cologne, University of Cologne, Cologne, Germany

^2^Department of Cardiology, Heart Center, Faculty of Medicine and University Hospital Cologne, University of Cologne, Cologne, Germany

^3^Philips Healthcare Germany, Hamburg, Germany

Corresponding author (*):

Jan Paul Janssen, MD

Institute for Diagnostic and Interventional Radiology, University Hospital Cologne

Kerpener Str. 62, 50937 Cologne, Germany

jan.janssen@uk-koeln.de

Phone: +49-221-478-82384

Fax: +49-221-478-82384

**Supplementary Table S1: Subjective ratings and shares of diagnostic image quality based on patient-level median ratings across readers excluding CE-MRA examinations with mistiming (n = 41)**

|  | **REACT** | **4D CE-MRA** | **Median of paired differences** (REACT – 4D CE-MRA)  **(95 %-CI)** | **p (Wilcoxon)** |
| --- | --- | --- | --- | --- |
|  | **Median [IQR]** | **Median [IQR]** |  |  |
| **Image quality** |  |  |  |  |
| Overall | 4.00 [3.17-4.33] | 3.33 [2.67-3.67] | 0.67 (–0.67 to 1.00) | **<0.001** |
| MPA | 4.00 [3.50-4.00] | 3.00 [3.00-3.00] | 1.00 (0.00 to 1.00) | **<0.001** |
| LPA | 4.00 [3.00-5.00] | 3.00 [3.00-4.00] | 0.00 (0.00 to 1.00) | **<0.001** |
| RPA | 4.00 [4.00-5.00] | 3.00 [3.00- 4.00] | 1.00 (0.00 to 1.00) | **<0.001** |
| **Motion artifacts** |  |  |  |  |
| Overall | 4.33 [4.00-5.00] | 3.33 [3.00-3.67] | 1.00 (1.00 to 1.33) | **<0.001** |
| MPA | 4.00 [4.00-5.00] | 3.00 [3.00-4.00] | 1.00 (1.00 to 2.00) | **<0.001** |
| LPA | 4.00 [4.00-5.00] | 3.00 [3.00-4.00] | 1.00 (1.00 to 1.00) | **<0.001** |
| RPA | 4.00 [4.00-5.00] | 3.00 [3.00-4.00] | 1.00 (1.00 to 1.00) | **<0.001** |
| **Susceptibility artifacts** |  |  |  |  |
| Overall | 4.67 [3.17-5.00] | 4.67 [3.67-5.00] | 0.00 (–0.33 to 0.00) | 0.060 |
| MPA | 4.00 [3.00-5.00] | 4.00 [3.00-5.00] | 0.00 (0.00 to 0.00) | 0.747 |
| LPA | 5.00 [3.50-5.00] | 5.00 [4.00-5.00] | 0.00 (0.00 to 0.00) | **0.008** |
| RPA | 5.00 [3.00-5.00] | 5.00 [4.00-5.00] | 0.00 (0.00 to 0.00) | 0.264 |
| **Shares of image quality ≥2** | REACT diagnostic [n, %] | 4D CE-MRA diagnostic [n, %] | Discordant b / c | p (McNemar) |
| Overall  (3/3) | 34/41, 82.9% | 36/41, 87.8% | 0 / 2 | 0.50 |
| MPA | 38/41, 92.7% | 38/41, 92.7% | 0 / 0 | 1.00 |
| LPA | 38/41, 92.7% | 39/41, 95.1% | 0 / 1 | 1.00 |
| RPA | 39/41, 95.1% | 41/41, 100% | 0 / 2 | 0.50 |

4D CE-MRA, 4-dimensional contrast- enhanced magnetic resonance angiography; LPA, left pulmonary artery; MPA, main pulmonary artery; REACT, relaxation-enhanced angiography without contrast and triggering; RPA, right pulmonary artery

**Supplementary Table S2: Pulmonary artery diameter measurements based on patient-level mean values across readers excluding CE-MRA examinations with mistiming**

| Segment | REACT  diameter  mean ± SD [mm] | 4D CE-MRA  diameter  mean ± SD [mm] | p  (t-test) | Bias (REACT – 4D CE-MRA)  [mm] | LoA (mm) | Outliers >3 mm  [n, %] |
| --- | --- | --- | --- | --- | --- | --- |
|  |  |  |  |  |  |  |
| MPA (n=38) | 25.41 ± 5.60 | 25.22 ± 5.46 | 0.58 | 0.19 | -3.82 to 4.19 | 8, 21.1% |
| LPA (n=38) | 18.63 ± 6.72 | 19.06 ± 6.66 | 0.17 | -0.43 | -4.09 to 3.23 | 5, 13.2% |
| RPA (n=39) | 17.38 ± 5.42 | 18.29 ± 5.26 | **<0.001** | -0.91 | -3.98 to 2.16 | 4, 10.5% |

4D CE-MRA, 4-dimensional contrast- enhanced Magnetic Resonance Angiography; REACT, relaxation-enhanced angiography without contrast agent and triggering; MPA, main pulmonary artery; LPA, left pulmonary artery; RPA, right pulmonary artery LoA; limits of agreement

**Supplementary Table S3: Subgroup analysis by implant type excluding CE-MRA mistiming cases**

| Group | n | Δ IQ median [IQR] | p (vs. 0) | Δ Motion median [IQR] | p (vs. 0) | Δ Susceptibility median [IQR] | p (vs. 0) |
| --- | --- | --- | --- | --- | --- | --- | --- |
| Stent | 15 | 0[-1-0] | >0.99 | 1[1-2] | **<0.001** | 0[-1-0] | 0.45 |
| CPV | 16 | 1[0-1] | **0.03** | 1[1-1.75] | **0.001** | 0[0-0] | 0.75 |
| None | 10 | 1[0-1.25] | **0.02** | 1[0-2] | **0.02** | 0[-0.25-0.25] | >0.99 |
| *Across-groups p (KW)* |  | ***0.02**** |  | *0.54* |  | *0.30* |  |
|  | n | REACT  diameter  mean ± SD [mm] | 4D CE-MRA  diameter  mean ± SD [mm] | p (paired t) | Bias (REACT – 4D CE-MRA) [mm] | LoA (mm) | Outliers >3 mm  [n,%] |
| Stent | 9 | 13.35 ± 4.53 | 13.73 ± 4.22 | 0.44 | -0.38 | -3.14 to 2.38 | 0, 0% |
| CPV | 16 | 23.33 ± 5.41 | 23.54 ± 5.48 | 0.65 | -0.21 | -3.76 to 3.34 | 3, 18.8% |
| None | 10 | 29.89 ± 4.66 | 29.48 ± 4.22 | 0.40 | 0.41 | -2.44 to 3.25 | 1, 10.0% |
| *Across-groups p (Welch’s ANOVA)* |  |  |  |  | *0.50* |  |  |
|  | n | REACT diagnostic [n, %] | 4D CE-MRA diagnostic [n, %] | Discordant b / c | p (McNemar) |  |  |
| Stent | 15 | 9, 60.0% | 11, 73.3% | 0 / 2 | 0.50 |  |  |
| CPV | 16 | 16, 100% | 16, 100% | 0 / 0 | 1.00 |  |  |
| None | 10 | 10, 100% | 10, 100% | 0 / 0 | 1.00 |  |  |

*Dunn's multiple comparisons test: stent vs. conduit/patch/valve *p* = 0.14; stent vs. none *p = 0.03*; conduit/patch/valve vs. none *p* > 0.99

4D CE-MRA, 4-dimensional contrast- enhanced magnetic resonance angiography; CPV, conduit/patch/valve; IQ, image quality; IQR, interquartile range; REACT, relaxation-enhanced angiography without contrast agent and triggering; LoA, limits of agreement
